# CBCT-based volumetric and dosimetric variation evaluation of volumetric modulated arc radiotherapy in the treatment of nasopharyngeal cancer patients

**DOI:** 10.1186/1748-717X-8-279

**Published:** 2013-12-01

**Authors:** Xiance Jin, Weigang Hu, Haijiao Shang, Ce Han, Jinling Yi, Yongqiang Zhou, Congying Xie

**Affiliations:** 1 Department of Radiotherapy and Chemotherapy, the 1st Affiliated Hospital of Wenzhou Medical University, Wenzhou 325000, China; 2 Department of Oncology, Shanghai Medical College, Fudan University, Shanghai, China; 3 Research department of IPTA(Beijing) Investment Adminstration Co.,Ltd, Beijing, China

**Keywords:** CBCT-based dose calculation, Deformable image registration, Volumetric modulated arc therapy, Nasopharyngeal cancer, Adaptive replanning

## Abstract

**Objective:**

To investigate the anatomic and dosimetric variations of volumetric modulated arc therapy (VMAT) in the treatment of nasopharyngeal cancer (NPC) patients based on weekly cone beam CT (CBCT).

**Materials and methods:**

Ten NPC patients treated by VMAT with weekly CBCT for setup corrections were reviewed retrospectively. Deformed volumes of targets and organs at risk (OARs) in the CBCT were compared with those in the planning CT. Delivered doses were recalculated based on weekly CBCT and compared with the planned doses.

**Results:**

No significant volumetric changes on targets, brainstem, and spinal cord were observed. The average volumes of right and left parotid measured from the fifth CBCT were about 4.4 and 4.5 cm^3^ less than those from the first CBCT, respectively. There were no significant dose differences between average planned and delivered doses for targets, brainstem and spinal cord. For right parotid, the delivered mean dose was 10.5 cGy higher (p = 0.004) than the planned value per fraction, and the V26 and V32 increased by 7.5% (p = 0.002) and 7.4% (p = 0.01), respectively. For the left parotid, the D50 (dose to the 50% volume) was 8.8 cGy higher (p = 0.03) than the planned values per fraction, and the V26 increased by 8.8% (p = 0.002).

**Conclusion:**

Weekly CBCTs were applied directly to study the continuous volume changes and resulting dosimetric variations of targets and OARs for NPC patients undergoing VMAT. Significant volumetric and dosimetric variations were observed for parotids. Replanning after 30 Gy will benefit the protection on parotids.

## 

Due to its sharp dose gradient, intensity modulated radiotherapy (IMRT) has been accepted as the primary treatment modality for nasopharyngeal cancer (NPC) patients [1,2]. Studies have confirmed that the dosimetric advantages of IMRT over conventional treatment translated into clinical outcomes with reduced parotid toxicity [3]. However, geometry and anatomic changes during the long course of IMRT treatment have limited the clinical benefits of IMRT [4].

Onboard cone beam CT (CBCT) has been applied to resolve the critical aspects of IMRT, such as patient setup and target localization [5]. The CBCT using a kilovoltage (kV) imaging system mounted on a linear accelerator has emerged as a significant technique for registering the soft tissue [6]. Anatomic changes in head and neck cancer patients throughout the radiation therapy treatment course due to tumor shrinkage, body weight loss, and soft tissue changes have been reported [7,8]. Daily CBCT for setup purposes during image-guided radiotherapy (IGRT) has been conducted to assess the soft tissue changes [9,10]. However, dosimetric variation and accuracy are more of concern during the radiotherapy course. The feasibility and accuracy of applying CBCT-based dose calculation are still under investigation due to the severe scatter problem of CBCT images [11].

Weekly computed tomography images during IMRT in the treatment of head and neck patients have been conducted to study the spatial variability and dosimetric differences between planned and delivered dose [12,13]. However, rescanning and replanning with weekly CT are not favored because of the time consuming and additional machine occupancy [14]. Weekly CBCT employed directly for dosimetric verification for IMRT or VMAT in the treatment of NPC patients is a promising solution.

In a previous study, we had achieved reasonable dose calculation accuracy for head-and-neck cancer patients based on CBCT with a region of interest (ROI) mapping method [15]. The purpose of this study is to evaluate the anatomic changes and related dosimetric effect based on weekly CBCT directly for NPC patients undergoing volumetric modulated arc therapy (VMAT) treatment.

## Materials and methods

### Patient characteristics and planning

This study was approved by the Institutional Review Board and performed at the 1st Affiliated Hospital of Wenzhou Medical University. We retrospectively reviewed 10 consecutive NPC patients treated by dual arc VMAT between January 2011 and November 2012 with weekly CBCT for setup error corrections. All the patients had diagnosed NPC with various AJCC stages, as summarized in Table 1. Five patients received induction chemotherapy with paclitaxel and cisplatin. One was treated by concurrent chemotherapy with paclitaxel, and the other four were treated by radiotherapy only. Patients were immobilized with a thermoplastic head mask and scanned on a planning kilovoltage CT scan (Philips Medical Systems, Eindhoven, The Netherlands) with a 3-mm slice thickness.

**Table 1:** Characteristics of NPC patients

**Patients**	**Sex**	**Age**	**Stages**	**Chemotherapy**	**GTV (cm**^**3**^**)**	**CTV (cm**^**3**^**)**
1	M	48	T4N1M0	NA	32.8	638.6
2	M	63	T2bN2M0	NA	92.8	668.0
3	M	71	T3N0M0	NA	89.7	652.1
4	M	59	T3N1M0	Induction	26.7	561.3
5	M	71	T2bN1M0	Induction	212.0	725.6
6	F	66	T1N1M0	Induction	93.1	603.8
7	M	55	T2bN1M0	Induction	37.6	443.0
8	M	39	T3N2M0	Concurrent	37.2	458.0
9	M	48	T2bN3bM0	Induction	28.2	719.4
10	M	62	T1N1M0	NA	21.4	299.6

Target and normal tissue delineations have been reported in our previous study and generalized here only briefly [16]. Gross tumor volume (GTV) was delineated as the mass shown in the enhanced CT images and/or MRI images, including the nasopharyngeal tumor, retropharyngeal lymphadenopathy, and enlarged neck nodes. The clinical target volume (CTV) was defined as the GTV plus a margin of potential microscopic spread, encompassing the inferior sphenoid sinus, clivus, skull base, nasopharynx, ipsilateral ppharyngeal space, and posterior third of the nasal cavity and maxillary sinuses. High-risk nodal regions, including the bilateral upper deep jugular nodes, submandibular nodes, jugulodigastric, mid-jugular, low jugular, and supraclavicular nodes and the posterior cervical nodes were included. The planning target volume (PTV) was created by adding a 3 mm margin to the CTV to account for setup variability. Prescription doses were 70 Gy and 56 Gy for GTV and CTV in 28 fractions, respectively. OARs consisting of the brainstem, spinal cord, left and right parotids were constrained for optimization.

Dual arc VMAT plans were generated on Philips Pinnacle^3^ treatment planning system (TPS) (clinical version 9.2; Philips, Fichburg, WI,USA). Optimization pmeters and process have been reported in our previous study [17]. Briefly, the first arc rotates clockwise with a start angle of 181° and a stop angle of 180°, and the second arc rotates counterclockwise from 180° to 181°. During the optimization, leaf motion of 0.46 cm/deg and a final arc space degree of 4 were employed.

### CBCT imaging and number to density curve calculation

VMAT plans were delivered on an Elekta Synergy linac (Elekta Ltd., Crawley, UK) which integrated an onboard kV-CBCT. CBCT images were acquired at the first treatment day (CBCT 1) with patients in the treatment position prior to radiation delivery, and then performed weekly after. The acquisition pmeters were 120 kV, 25 mA, 40 ms per projection with F0 filter. A total of about 650 projections were acquired for a full rotation in about 2 min. S20 collimator cassette was used on all patients giving a nominal irradiated scan length at the isocenter of approximately 26 cm.

**Figure 1:**
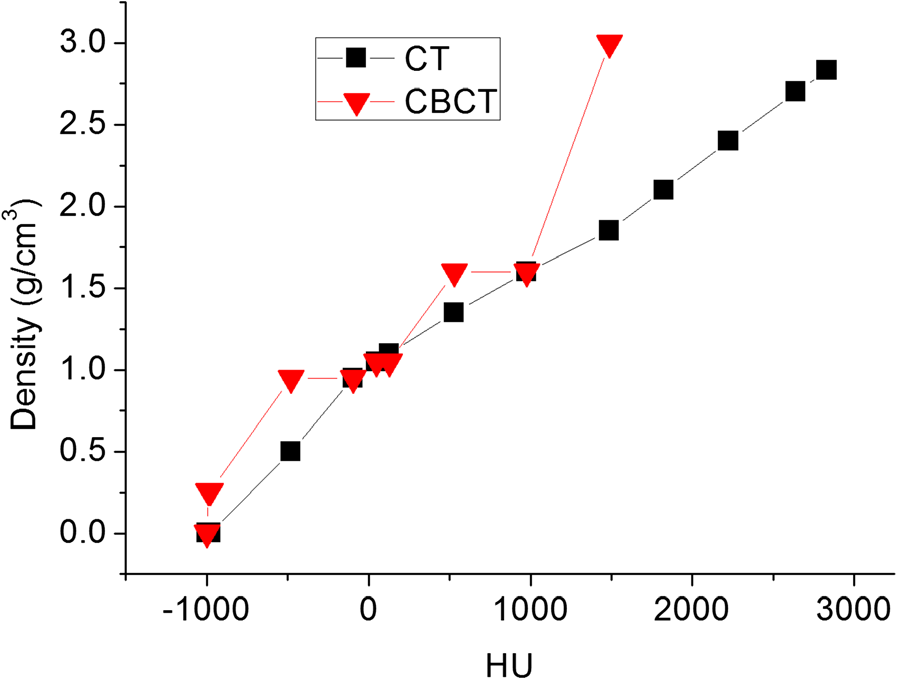
HU to density calibration curves for CT and CBCT.

A region of interest (ROI) CT number mapping method was used to generate the CBCT number to physical electron density conversion curve for the dose calculation with a phantom, Catphan-600 module CTP503 (Phantom Laboratory, NY) [18]. This process has been reported in our previous study [15] and summarized here: (1) register the planning CT images and kV-CBCT images in the Pinnacle TPS; (2) map the ROIs from conventional CT dataset to the CBCT dataset, and record the mean CBCT number values of these ROIs, and (3) Generate the kV-CBCT numbers to physical electron density calibration curve based on the density values measured on the conventional CT. The typical CT number to density curves for CT and CBCT were presented in Figure 1.

### Volumetric and dosimetric evaluation

Volumetric changes and resulting dosimetric effects based on CBCT images were investigated using the Raystation TPS (version 3.5, RaySearch, Stockholm, Sweden). The Raystation TPS was commissioned with the same beam data as the Pinnacle system. The dose deviations between Raystation and Pinnacle were within 1.5% during the commission process. All VMAT plans with initial CT data were exported from Pinnacle TPS to Raystation TPS through DICOM service and the dose distributions were recalcuated based on the same CT number to density calibration curve. Weekly CBCT images were also imported into the Raystation TPS through DICOM service. For each patient, each weekly CBCT image was rigidly registered to the planning CT individually. The rigid registration was performed automatically and final manual adjustment was used for better alignment. After the rigid registration, a deformable registration was also performed automatically using vertex-vertex correspondence between the reference image set and the target image sets. That is the user can convert an region of interest (ROI) with contour shape to a new ROI with triangle mesh shape. The new ROI can be used as controlling ROI, which means that it has the same number of vertices in all image sets and that it has point-to-point correspondence for the vertices. As a result, each weekly CBCT image had one rigid and one deformed registration to the original planning CT. Auto contours were conducted for target volumes and OARs on weekly CBCTs by mapping the contours in the planning CT to CBCT with the deformable registrations. A physician carefully evaluated all contours and corrections were performed if necessary.

For each patient, the beam arrangements and optimization pmeters in the initial treatment plan on the planning CT was directly applied to the weekly CBCTs. Using the CBCT number to density calibration curve, the fractional dose based on the weekly CBCT were recalculated. To compare the planned dose in the initial planning CT and the delivered dose on weekly CBCT, the dose to 95% (D95) and 90% (D90) of the GTV and CTV, and the volume of CTV irradiated by 110% of the prescription dose (V110) were recorded and compared. The dose to 1% (D1) of brainstem and spinal cord, the dose to 50% (D50) of parotids, the mean dose (Dmean), the volume of parotids receiving 26 Gy (V26) and 32 Gy (V32) were also recorded and compared.

### Statistical analysis

Descriptive statistics were calculated to characterize the dosimetric and volumetric changes of targets and OARs. Comparisons between the planned dose in the initial CT and recalculated dose based on weekly CBCTs were analyzed using one-way ANOVA. When an overall significant difference was observed, the post hoc Tukey test was used to determine which pairwise comparisons differed. All statistical analyses were conducted with SPSS 17.0 software (spss Inc., Chicago, IL). Differences were considered statistically significant when p < 0.05.

## Results

**Figure 2:**
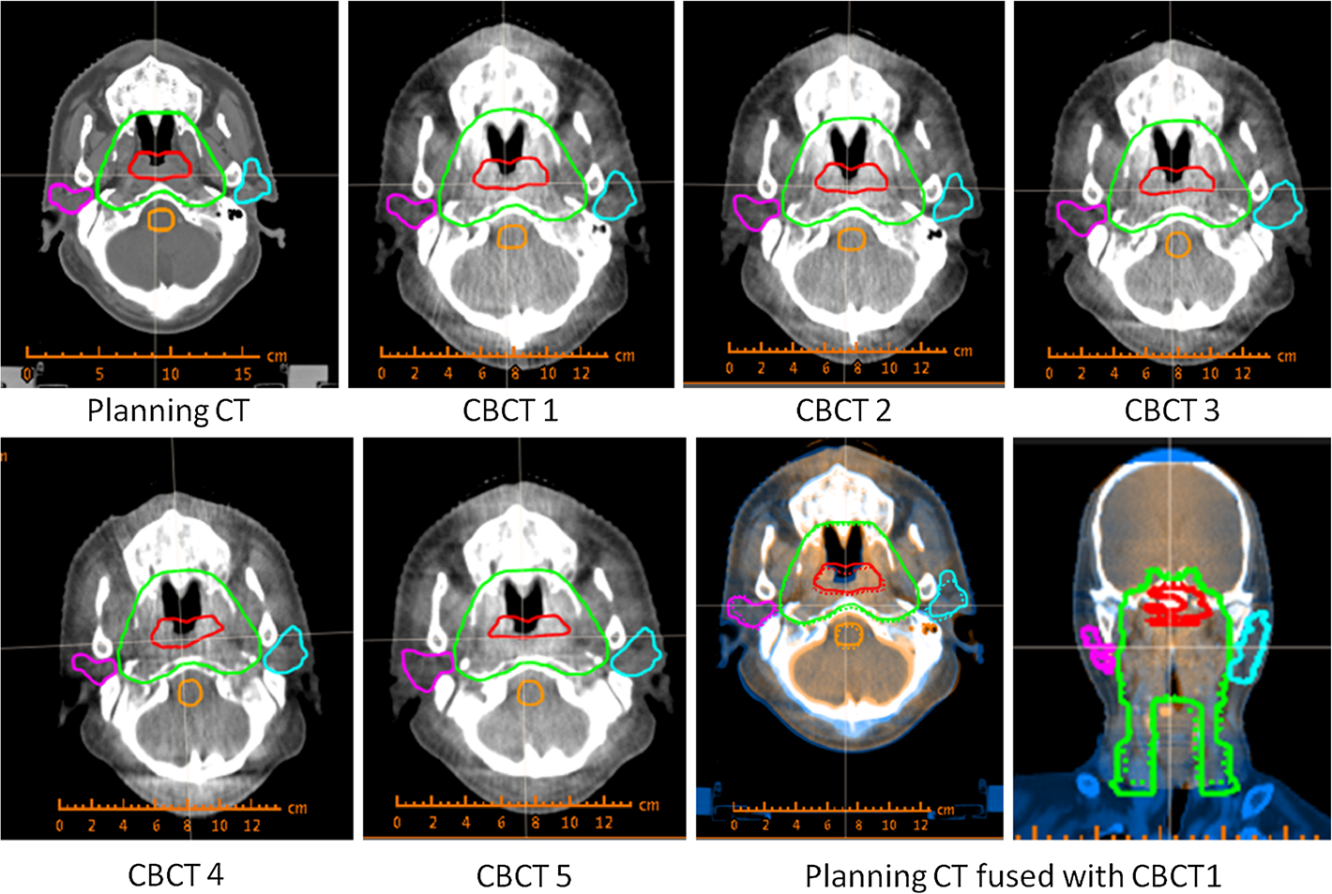
Planning CT with manual contours, CBCTs with deformed contours and a typical fusion image.

**Figure 3:**
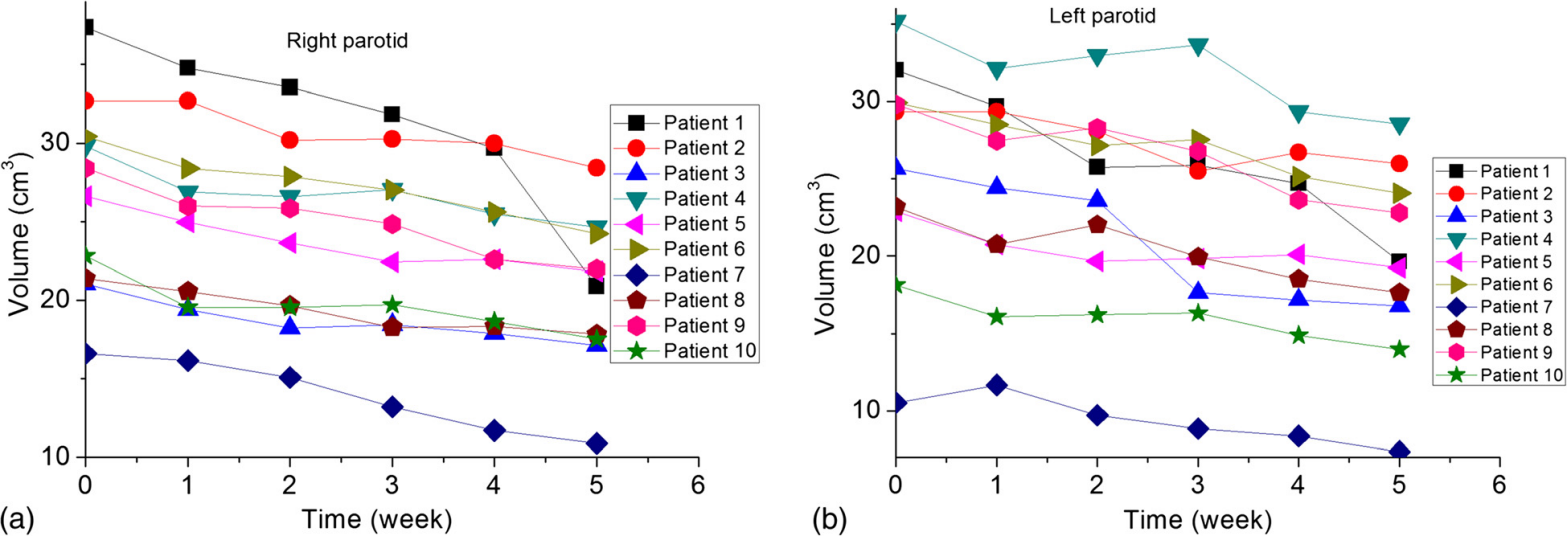
Volume changes measured with weekly CBCT of a) right parotid and b) left parotid.

Figure 2 shows the typical planning CT with manual contours and the weekly CBCT with deformed contours. Detailed average volume changes for targets and OARs were listed in Table 2. The average volumes of CTV on the first CBCT were smaller than those in the planning CT, however, no statistical difference of volume changes were observed during the treatment course. There were also no general trend and significant volume changes for GTV and brainstem. The average volumes of parotids on the first CBCT (CBCT 1) before the radiotherapy were also close to those in the planning CT, but the average volumes of parotids decreased continuously during the treatment course. The average volume of the right parotid and left parotid measured from the fifth CBCT (CBCT 5) were about 4.4 and 4.5 cm^3^ less than those measured from the CBCT 1, respectively. Individual volume changes for both parotids were presented in Figure 3.

**Table 2:** Average volume changes from the planning CT to the CBCTs

**Volume (cm**^**3**^**)**	**GTV**	**CTV**	**Brainstem**	**Cord**	**Right parotid**	**Left parotid**
Planning CT	67.1 ± 58.7	577.0 ± 137.5	27.7 ± 2.3	13.6 ± 4.7	26.7 ± 6.3	25.7 ± 7.3
CBCT 1	66.4 ± 59.2	512.3 ± 130.9	27.5 ± 2.4	12.5 ± 2.2	24.9 ± 6.9	24.1 ± 6.6
CBCT 2	66.5 ± 58.6	525.2 ± 138.7	28.5 ± 3.7	13.6 ± 5.0	24.0 ± 5.8	23.3 ± 6.8
CBCT 3	66.0 ± 57.5	520.6 ± 141.7	27.4 ± 2.3	12.9 ± 3.3	23.3 ± 5.9	22.2 ± 7.1
CBCT 4	64.4 ± 57.0	500.9 ± 125.0	26.9 ± 2.3	12.6 ± 2.2	22.3 ± 5.7	20.9 ± 6.3
CBCT 5	65.9 ± 57.8	506.6 ± 137.5	26.8 ± 3.5	13.8 ± 3.6	20.5 ± 4.9	19.6 ± 6.2

Dosimetric differences resulting from volume changes and geometrical errors were summarized in Table 3. There were no significant dose differences between the average planned dose and recalculated delivered dose for both GTV and CTV. There were also no significant differences to the average maximum dose of brainstem and spinal cord between planned and delivered doses.

**Table 3:** Dosimetric differences between planned dose and delivered dose calculating from CBCT

	**Planned dose**	**CBCT1**	**CBCT2**	**CBCT3**	**CBCT4**	**CBCT5**	**p**
GTV							
D95 (cGy)	228.6 ± 3.8	230.1 ± 5.0	227.8 ± 6.8	226.6 ± 6.3	228.5 ± 7.1	230.3 ± 9.9	0.83
D90 (cGy)	233.2 ± 3.6	235.0 ± 5.1	231.6 ± 7.5	224.6 ± 23.3	232.9 ± 7.1	227.1 ± 23.4	0.57
CTV							
V110	29.3 ± 13.0	32.0 ± 14.0	28.9 ± 14.3	28.8 ± 13.9	30.3 + 14.7	30.1 + 14.2	1.00
D95 (cGy)	188.6 ± 3.0	189.9 ± 4.5	188.1 ± 4.7	187.5 ± 4.5	188.0 ± 4.5	189.1 ± 5.0	0.86
D90 (cGy)	192.8 ± 2.7	194.5 ± 3.7	192.7 ± 5.4	193.1 ± 4.9	193.0 ± 4.8	194.6 ± 3.3	0.83
D1 (cGy)							
Brainstem	153.6 ± 11.2	150.1 ± 11.2	150.1 ± 15.8	151.6 ± 17.4	149.7 ± 16.6	152.1 ± 16.8	1.00
Cord	130.3 ± 10.4	130.3 ± 6.3	133.7 ± 16.2	133.4 ± 11.6	130.5 ± 13.6	137.3 ± 16.0	0.79
Right parotid							
D50 (cGy)	74.5 ± 3.4	79.9 ± 5.0	77.8 ± 4.5	81.6 ± 5.3	82.8 ± 6.2	82.2 ± 3.7	0.002
Dmean (cGy)	95.6 ± 5.4	101.9 ± 5.3	99.1 ± 6.4	102.6 ± 6.8	106.1 ± 8.0	101.2 ± 4.4	0.012
V26	36.5 ± 3.0	41.5 ± 3.8	38.9 ± 4.3	41.3 ± 5.0	44.0 ± 4.5	42.3 ± 3.5	0.003
V32	29.3 ± 4.9	33.7 ± 4.7	31.2 ± 5.2	35.3 ± 5.2	36.7 ± 4.6	36.0 ± 4.0	0.006
Left parotid							
D50 (cGy)	74.9 ± 2.6	80.0 ± 3.7	77.3 ± 5.7	76.4 ± 9.2	83.7 ± 7.3	82.0 ± 6.3	0.017
Dmean (cGy)	94.8 ± 8.1	101.4 ± 10.2	99.6 ± 8.2	101.9 ± 7.6	104.6 ± 9.8	103.3 ± 7.6	0.18
V26	34.8 ± 3.3	39.8 ± 5.3	39.0 ± 2.7	40.5 ± 5.5	43.6 ± 6.4	42.9 ± 4.9	0.02
V32	27.5 ± 5.5	31.2 ± 6.7	31.2 ± 5.7	32.7 ± 5.9	35.1 ± 7.2	33.6 ± 7.6	0.17

Dose delivered to the parotids demonstrated some significant differences. Detailed pairwise comparison p values between planned dose and recalculated CBCT dose for parotids were presented in Table 4. As presented in Table 3 and 4, the D50 of right parotid increased significant since CBCT 3 at the 10th fraction with a dose of 7.1 cGy higher than (p = 0.02) planned dose per fraction. The mean dose of right parotid was 10.5 cGy higher than (p = 0.004) planned dose per fraction after CBCT 4 at the 15th fraction. The V26 and V32 of right parotid from CBCT 4 increased by 7.5% (p = 0.002) and 7.4% (p = 0.01) compared to the planned values, respectively. The D50 of left parotid was 8.8 cGy higher than (p = 0.03) planned dose per fraction from CBCT 4. The V26 of the left parotid from CBCT 4 increased by 8.8% (p = 0.002) compared to the planned value.

**Table 4:** Comparison between planned parotid dose and delivered parotid dose calculated using CBCT

**p**	**Planned vs CBCT 1**	**Planned vs. CBCT 2**	**Planned vs. CBCT 3**	**Planned vs. CBCT 4**	**Planned vs. CBCT 5**
Right parotid					
D50 (cGy)	0.13	0.64	0.02	0.004	0.01
Dmean (cGy)	0.22	0.80	0.13	0.004	0.34
V26	0.09	0.78	0.11	0.002	0.03
V32	0.32	0.94	0.07	0.01	0.03
Left parotid					
D50 (cGy)	0.45	0.95	1.00	0.03	0.13
V26	0.21	0.38	0.11	0.002	0.01

## Discussion

Anatomic and dosimetric variations of NPC in radiotherapy have long been concerns. In this study, relying on the CBCT-based dose calculation, weekly CBCTs were applied directly to study the volumetric changes and resulting dosimetric effects of 10 consecutive NPC patients underwent VMAT treatment.

Due to the limited field of view, the CBCT may not span the complete longitudinal dimension of the target volume for some NPC patients. The calculation grids in the initial planning CT of two patients were adjusted and shortened in longitudinal direction to match the target volumes in the initial CTs and in the CBCTs. The average volumes of CTV and spinal cord on the first CBCT were smaller than those in the planning CT. However, except for parotids, no significant volume changes of targets and OARs were observed based on CBCT 1 in this study. The volume changes of parotids were unique for each individuals as shown in Figure 3. According to Table 2, the average weekly shrinkage of right and left parotids were 4.4% and 4.7%, respectively. This was close to the reported glands shrinkage of 4.9%/wk in the study of Robar et al., in which weekly CT was applied to study the spatial variability of OAR and the resultant dosimetric effects during IMRT for 15 head and neck patients [12].

Currently, the design of onboard CBCT is far from optimal and its quality is adversely influenced by many factors, such as scatter, beam hardening and intra-scanning organ motion. The question of whether CBCT images can be used directly for radiation dose calculation has been raised and investigated. Based on reliable CBCT HU and density calibration curve, studies have demonstrated the reliability and accuracy of CBCT-based dose calculation [19,20]. Our previous study also demonstrated that ROI mapping method was an effective and simple method for the CBCT-based dose calculation [15]. Therefore, we applied the same method in this study to investigate the dosimetric effects during VMAT based on weekly CBCT directly.

There were no significant differences between the planned and delivered doses for GTV and CTV. This was consistent with the study of Zhang et al., in which a planning CT and weekly repeat CT were scanned to study the actual dose variability of targets and OARs for 11 NPC patients during IMRT [21]. The average maximum doses represented by D1 for the brainstem and spinal cord were not significant different between planned and delivered doses. However, patient 1 demonstrated a 16.8% and 26.0% dose increase for brainstem and spinal cord on CBCT 5, respectively. This indicated a random dosimetric variability for brainstem and spinal cord similar to the reported results in the study of Robar et al. [12]. The increased dose on CBCT 5 could be caused by a dramatic anatomic changes resulting from a sharp volume shrinkage of parotids, as shown in Figure 3.

Both parotids shifted towards a greater delivered dose during the VMAT treatment, which was consistent with the studies based on weekly CT during IMRT treatment [12,21]. Compared to the planned dose, the delivered dose on D50, Dmean, V26 and V32 of parotids were increased significantly after CBCT 4, which was obtained at the 15th fraction with a dose of 30 Gy and 37.5 Gy for CTV and GTV, respectively. This implies a replanning after 30 Gy will benefit the parotid protection for NPC patients during VMAT treatment. However, the time trend for the dosimetric changes of parotids was less obvious compared to the time trend of their volume changes, as shown in Table 3. The small dosimetric fluctuations among CBCTs could be caused by the errors of CBCT based dose calculation [15]. Another explanation could be the limited number of patients included in this study. Additional work on a larger patient population is warranted to decide the proper adaptive replanning time for NPC patients.

CBCT and megavoltage CT (MVCT) [4] have been widely employed during the radiotherapy for geometric error corrections. Direct dose calculation based on CBCT and MVCT will certainly provide a more convenient and straightforward way than weekly repeated CT images for adaptive replanning [12,13]. However, due to the intrinsic limitations of CBCT, extensive work on the reliability and accuracy of CBCT-based dose calculation is also warranted to evaluate whether our findings were accurate enough and could actually translate into guidelines.

## Conclusion

CBCT-based dose calculation was applied directly to study the anatomic changes and resulting dosimetric variations for NPC patients undergoing VMAT treatment. Continuous volume changes in the parotids were observed with weekly CBCT. Significant dosimetric variations in the parotids were presented at 15th fraction with CBCT 4. No significant difference in volumetric and dosimetric variations were observed for other OARs and targets. A replanning after 30 Gy may be useful in the VMAT for NPC.

## Consent

Written informed consent was obtained from the patient for the publication of this report and any accompanying images.

## Competing interests

The authors declare that they have no competing interests.

## Authors’ contributions

Each author has participated sufficiently in the work to take public responsibility for appropriate portions of the content. XJ, CX designed the study. WH did the CBCT calculation, HS and YJ performed the study and analysis. YZ provided the patients’ images. The manuscript was written by XJ, all other authors helped and finally approved the final manuscript.
